# Calcium pre-conditioning substitution enhances viability and glucose sensitivity of pancreatic beta-cells encapsulated using polyelectrolyte multilayer coating method

**DOI:** 10.1038/srep43171

**Published:** 2017-02-27

**Authors:** Niusha Nikravesh, Sophie C. Cox, Gurpreet Birdi, Richard L. Williams, Liam M. Grover

**Affiliations:** 1 School of Chemical Engineering, University of Birmingham, Birmingham, B15 2TT, UK

## Abstract

Type I diabetics are dependent on daily insulin injections. A therapy capable of immunoisolating pancreatic beta-cells and providing normoglycaemia is an alternative since it would avoid the late complications associated with insulin use. Here, 3D-concave agarose micro-wells were used to culture robust pancreatic MIN-6 cell spheroids within 24 hours that were shown to exhibit cell-cell contact and uniform size (201 ± 2 μm). A polyelectrolyte multilayer (PEM) approach using alginate and poly-l-lysine was employed to coat cell spheroids. In comparison to conventional PEM, use of a novel Ca^2+^ pre-coating step enhanced beta-cells viability (89 ± 6%) and metabolic activity since it reduced the toxic effect of the cationic polymer. Pre-coating was achieved by treating MIN-6 spheroids with calcium chloride, which enabled the adhesion of anionic polymer to the cells surface. Pre-coated cells coated with four bilayers of polymers were successfully immunoisolated from FITC-mouse antibody and pro-inflammatory cytokines. Novel PEM coated cells were shown to secret significantly (P < 0.05) different amounts of insulin in response to changes in glucose concentration (2 vs. 20 mM). This work presents a 3D culture model and novel PEM coating procedure that enhances viability, maintains functionality and immunoisolates beta-cells, which is a promising step towards an alternative therapy to insulin.

The encapsulation of cells within a polymeric semi-permeable membrane is attractive for various biomedical applications. In particular, this method has been studied to treat endocrine diseases, such as diabetes^1^. Type 1 diabetes, also known as diabetes mellitus, is an autoimmune disease that results from the failure in glucose regulation due to the destruction of pancreatic beta-cells by immune cells^2^. Typically insulin therapies are employed, however, continuous unregulated blood glucose levels can lead to a variety of secondary complications including, cardiovascular disease, blindness, kidney disease, and death^2^,^3^. Maintaining normoglycaemia would prevent such complications and improve patient’s quality of life^1^. A promising alternative therapy is to encapsulate beta-cells so that when transplanted the cells are protected from the host immune system, which would eliminate the need for immunosuppressant drugs. Critically this should be balanced with the diffusion of oxygen, signalling molecules, nutrients, and secreted products, such as insulin^2^,^4^.

Encapsulation of mammalian cells was first described by Lim and Sun, who formed alginate hydrogel microcapsules embedded with pancreatic islets^5^. Since then, the clinical application of this method has been hampered by various issues, such as poor revascularisation of the constructs after implantation, relatively large diameter microcapsules (400–800 μm) in comparison to the transplantation site, and an unfavourable ratio between encapsulated cells volume and overall capsule volume^6^,^7^. The two latter obstacles are related to the large distance between the encapsulated cells and the surrounding environment, which results in limited mass transfer, hypoxia, and ultimately cell dysfunction and death^1^.

A possible solution may be to coat cells with polyelectrolytes rather than embedding cells in a polymeric matrix. This technique, known as layer-by-layer (LBL) or polyelectrolyte multilayer coating (PEM), is based on the alternate deposition of anionic and cationic polymers on to a charged surface^8^,^9^. This approach limits the gap between the cells and the surrounding environment resulting in a shorter response time to external stimulation^10^, while retaining a coating that may prevent immune response. This may allow rapid response to changes in blood glucose and thereby better regulation.

Alginate is the most common and studied material for encapsulation of living cells and therapeutic agents^11^. Alginate is an anionic polymer that can form polyelectrolyte complexes in the presence of polycations, such as poly-l-lysine (PLL) and chitosan. PLL has been used to coat alginate beads as a way of controlling the ingress of biological components harmful to cell survival^12^,^13^. Owing to the negative charge of cells, PEM coating is initiated by the deposition of a cationic polymer, however, previous studies have shown that when cationic polymers are used in direct contact with cells it increases the possibility of host inflammatory responses and cytotoxic behaviour^1^,^14^.

In this study, a novel pre-coating step was introduced into a conventional PEM coating method to minimise the influence of PLL on cell viability by conditioning the surface of cell aggregates with CaCl_2_, before exposure to PLL. The resulting spheroids were analysed with respect to viability, functionality, and immunoisolation.

## Results

### Formation of uniform MIN-6 spheroids

**Figure 1:**
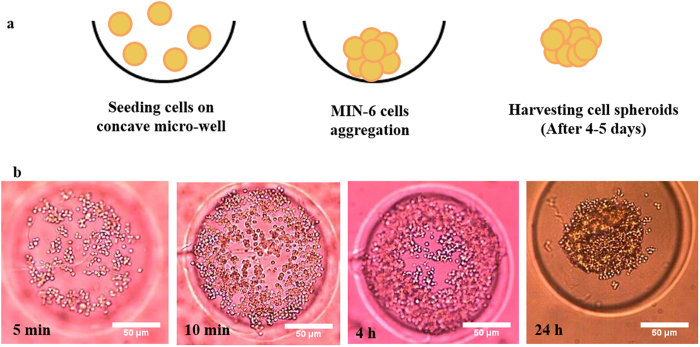
Formation of uniform MIN-6 spheroids. (**a**) Schematic illustration of cell aggregation within agarose-based micro-wells. (**b**) Light microscopy imaging of cell aggregation in wells during time intervals: 5, 10 min, 4, and 24 h of post-seeding.

To achieve uniform aggregation of pancreatic beta-cells prior to the PEM coating process, dispersed MIN-6 cells were seeded on top of agarose-based micro-wells. Cell spheroids with uniform size and shape (92 ± 4.9 μm) were generated in the centre of the concave wells within 24 h due to aggregation of each cell by cell-cell contact and gravitational force (Fig. 1b). The majority of cell spheroids formed after one day of culture and only a few micro-wells remained vacant (<1.5%). It was discovered that 4–5 days of culture was the optimum time period to achieve robust spheroids, which could be easily harvested and used for the coating procedure.

### MIN-6 spheroid morphology

**Figure 2:**
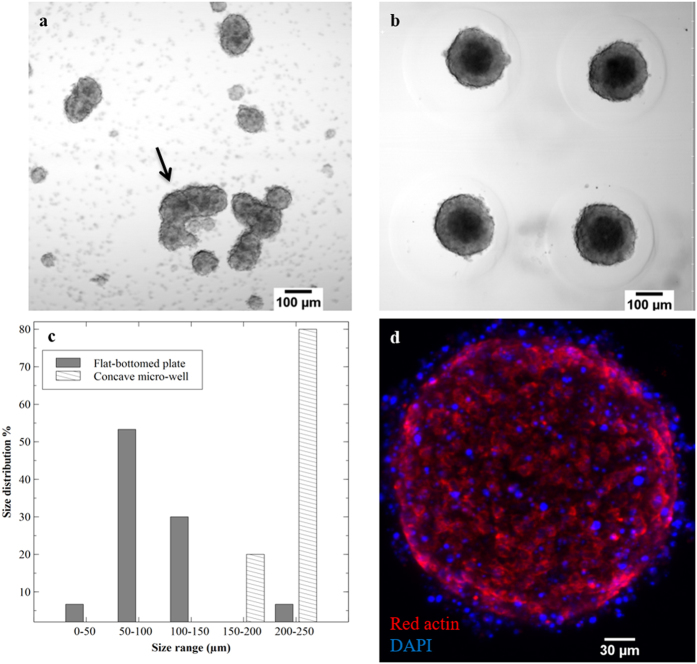
Comparison between MIN-6 spheroids cultured on different surfaces. (**a**) and (**b**) Morphology of MIN-6 cells cultured on flat-bottomed plate and agarose concave micro-wells on day 7, respectively. (**c**) Comparison between the size distribution of aggregated cells in both flat-bottomed and concave wells (n = 30 spheroids), large chain of cell clumps formed in flat-bottomed sample were not analysed in this study (Indicated with an arrow). (**d**) Direct immunofluorescent staining of MIN-6 spheroids with Actin-Red 555 (F-actin probe) and DAPI (cell nuclei probe) for visualisation of cell-cell interaction using CLSM.

Then to quantify the effect of culturing cells on 3D agarose constructs, the size distribution of 30 spheroids cultured on both a flat-bottomed plate (control) and concave wells were compared after 7 days of culture. In the case of MIN-6 spheroids in agarose-based wells, their size range was mostly between 200–250 μm with an average of 201 ± 2 μm. However, for the control sample, cells exhibited a wide range of sizes from 50–250 μm with an average of 103 ± 49.2 μm (Fig. 2a,b,c). To gain insight in to the influence of 3D culture, immunofluorescent staining of actin filaments and cell nuclei were examined on day 5. As expected, fluorescence signals from the filamentous actin (F-actin) cytoskeleton, suggested that the MIN-6 spheroids were adhered to one another, forming a compact structure. DAPI staining showed a large number of freely growing cells on the surface of the MIN-6 spheroids (Fig. 2d).

### Addition of pre-coating step increased cytocompatiblility of coated cells

**Figure 3:**
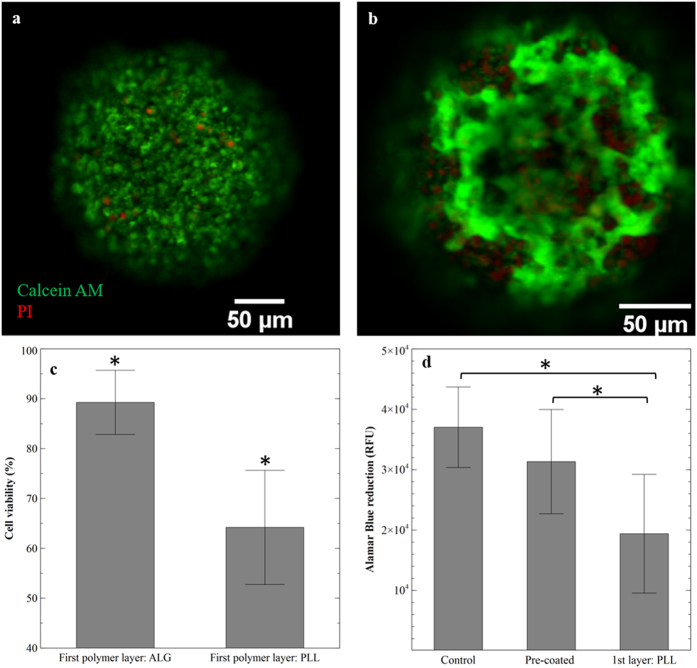
Evaluation of viability and metabolic activity of MIN-6 spheroids coated with four bilayers of PLL/ALG. (**a**) Confocal microscopic images of live (calcein-AM: green) and dead (PI: red) stained cell spheroids coated with initial layer of alginate during the pre-coating step. (**b**) Cells coated with PLL as the first coating layer. (**c**) Quantitative evaluation of live/dead analysis (n = 10 spheroids) (**d**) Comparison between metabolic activity of MIN-6 coated spheroids with and without pre-coating step, and the control (non-coated spheroids) (n = 3), Results are presented as mean ± S.D, (*P < 0.05).

Coating of MIN-6 spheroids was achieved by subsequent layering of oppositely charged polyelectrolytes; ALG (alginate) and PLL (poly-l-lysine). After the pre-coating step (pre-conditioning with CaCl_2_ and deposition of initial ALG layer), each coated spheroid was formed by the alternate deposition of four bilayers (a total of 8 layers) of polyelectrolytes. For all cell spheroids, ALG was chosen as the final polyelectrolyte layer. To investigate whether the pre-coating step was efficient in reducing the toxic effect of PLL, the viability of the pre-coated cells was compared with MIN-6 spheroids coated directly with PLL. After 8 days of culture, quantitative analysis of the stained cells revealed a significant difference (p < 0.01) between the viability of cells that were pre-coated (89 ± 6%) and those with an initial layer of PLL (64 ± 11%) (Fig. 3c).

Furthermore, the metabolic activity of coated MIN-6 spheroids (with and without the pre-coating step) was compared with non-coated cells as a control (Fig. 3d). In comparison to pre-coated spheroids and non-coated cells, the ability of non-pre-coated spheroids to reduce the Alamar Blue reagent (metabolic activity indicator) was significantly reduced (p = 0.04) after 9 days of culture, possibly due to the toxicity of PLL when used in direct contact with MIN-6 cells; Hence in comparison to the control, the metabolic activity of cells coated with an initial layer of PLL was reduced by 32%, while for the cells with pre-coating step there was a smaller reduction of 13%.

### Confirmation of coating on cell spheroid surfaces

**Figure 4:**
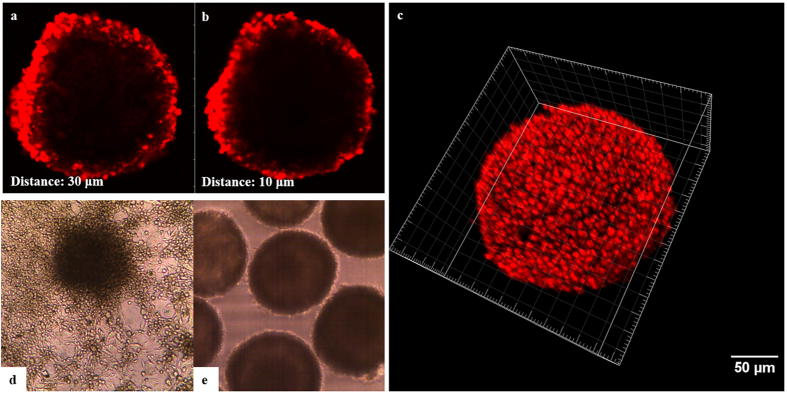
Monitoring the ALG/PLL coating layers on MIN-6 spheroids. (**a**) and (**b**) Z-stack cross-sections of coated MIN-6 spheroids with FITC-PLL obtained at 10 and 30 μm from the centre, respectively. (**c**) 3-D reconstruction of coated spheroid (Raw data from Z-stack was analysed with Imaris software). (**d**) and (**e**) Light Micrographs of coated and non-coated cell spheroids in response to cell-attachable surface, respectively.

To demonstrate the deposition of polyelectrolytes on cell spheroids, PLL labelled with Alexa Fluor-647 was used. The FITC labelled PLL was then applied to the final bilayer (7^th^ layer) to enable visualisation of coated cells under confocal microscope. From cross sectional images obtained at distances of 10 and 30 μm from the centre of cell spheroid, using Z-stack imaging technique (Fig. 4a,b), the fluorescent signalling was mainly located at the peripheries of the coated cells, which indicated successful coating of cells with alternate deposition of polyelectrolyte layers. Also Fig. 4d and e demonstrate the difference in behaviour of coated and non-coated spheroids when cultured on a CellBIND tissue culture plate. After 7 days of culture, non-coated spheroids attached to the surface and cells covered and migrated across the whole area, as expected. On the contrary, coated spheroids with 4 bilayers remained suspended in the culture medium confirming that the entire spheroid surface was coated.

### Coated MIN-6 cells excluded FITC-antibody

**Figure 5:**
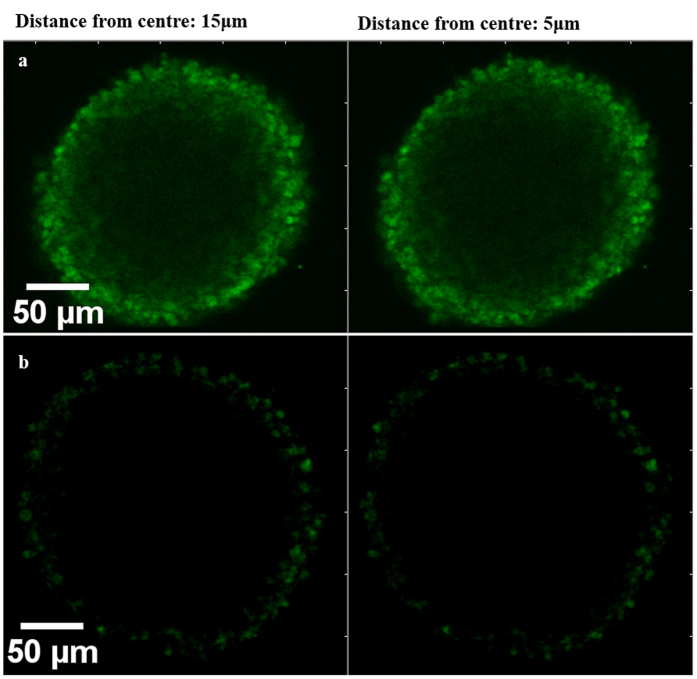
Confocal images of MIN-6 spheroids after being exposed to FITC-mouse antibody (10 KDa). (**a**) Z-stack image of non-coated spheroid using argon laser. (**b**) Coated spheroid with pre-coating step and 4 bilayers of PLL/ALG. Z-stack images were obtained at distance of 5 and 15 μm from the centre of both coated and non-coated spheroids.

To investigate the permeability of coated MIN-6 spheroids to immunologically relevant molecules, samples were exposed to FITC-mouse antibody which can then target the antigens (MHC class II) that exist on the surface of pancreatic beta-cells. The fluorescence intensity emitted from non-coated spheroids was shown to be much greater than coated spheroids (Fig. 5). This study provided evidence that compared with the control (non-coated cells), pre-coating of cells with alginate followed by the deposition of four bilayers of PLL and ALG considerably protected the coated pancreatic beta-cells from attaching to the FITC-antibody by covering the surface of spheroids.

### Coated cells exhibited higher survival after being exposed to cytokines

**Figure 6:**
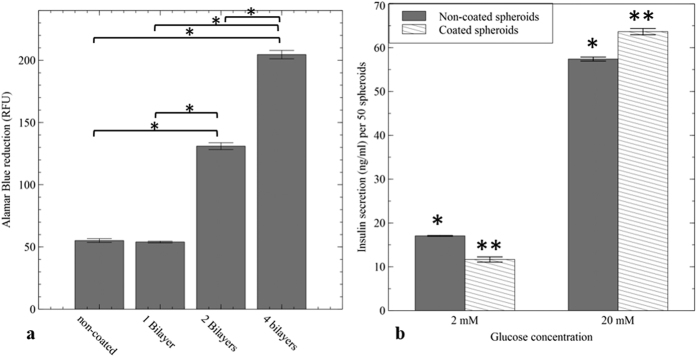
(**a**) Viability of non-coated and coated MIN-6 spheroids with different numbers of PLL/ALG bilayers after being incubated with a cytokine mixture for 24 h; Results are presented as mean ± SEM (n = 4), (*P < 0.01), and were normalised to 1000 cells. (**b**) Insulin release of non-coated (control) and coated MIN-6 spheroids in response to glucose stimulation. Insulin release was normalized against 50 spheroids; Results are presented as mean ± S.D (n = 3), (*, **P < 0.05).

To further examine the permeability of coated spheroids against smaller molecules, a mixture of three cytokines: IL-1β (~17 KDa), TNF-α (~17.5 KDa), and IFN-γ (~15 KDa) was added to both non-coated and coated spheroids with 1, 2, and 4 bilayers of polyelectrolytes. As shown in Fig. 6a, after 24 h exposure to cytokines, the viability and metabolic activity of non-coated cell spheroids were notably reduced to 55 ± 2.5 RFU (P-value < 0.01). Moreover, response of MIN-6 spheroids to cytokines after the addition of one bilayer of polyelectrolytes was only slightly different from the non-coated cells. However, deposition of four bilayers of PLL/ALG significantly reduced cytokine damage on pancreatic beta-cells survival rate (204 ± 3.3 RFU) in comparison with other groups (P-value < 0.01).

### Coated spheroids remained responsive to the changes in glucose concentrations

After two weeks of culture, insulin secretion from coated and non-coated MIN-6 spheroids (n = 50) in response to glucose was investigated by culturing both samples in a series of basal (2 mM), and stimulatory (20 mM) glucose concentrations. The amount of insulin released from coated spheroids into basal glucose solution was 17.1 ± 0.1 ng/mL, which was significantly increased to 57.3 ± 0.4 ng/mL under a stimulatory glucose concentration (P-value < 0.05) (Fig. 6b). Also, comparison between the amounts of secreted insulin from both coated and non-coated spheroids showed no significant difference, indicating that the coated MIN-6 cells remained responsive to glucose after applying four bilayers of ALG/PLL.

## Discussion

In this study, we have demonstrated the formation of stable and uniformly sized MIN-6 spheroids using agarose-based concave micro-wells. The prepared spheroids were then immunoisolated with ALG and PLL using a novel coating approach. Up to now, several groups have developed polydimethylsiloxane-based micro-molds for creating cell aggregates, however, using this substrate requires further steps for modification of well surfaces and preventing cell adhesion^15^,^16^,^17^. Herein, the formed beta-cell spheroids were easily detached and retrieved from the agarose construct without any further need for treatment with trypsin or surface modification, which was an advantage for preventing damage to cells during the harvesting and coating process.

Microscopic observation followed by size analysis confirmed that the use of concave micro-wells facilitated the formation of uniform MIN-6 spheroids with a size range similar to pancreatic islets (150–250 μm) (Fig. 2c). In contrast, the diameter of aggregated cells formed on a flat-bottomed plate varied between 50 and 250 μm, and also resulted in accumulation of cell chains with non-uniform shape and a broad size distribution (Fig. 2a). The viability of these samples was notably affected and therefore such shapes are undesirable for coating. Previous studies have shown that culturing epithelial cells in a 3D environment could improve cell adhesion and intracellular signalling by providing an additional dimension for external cell interactions^18^. As a result, formation of beta-cell spheroids were studied as a means to mimic the real tissue and cell adhesions were observed by direct fluorescent staining of F-actin and cell nuclei of MIN-6 spheroids (Fig. 2d).

For the past two decades, coating mammalian cells using a layer-by-layer assembly of polyelectrolytes has been studied as a promising approach to reduce large void volume between the encapsulated cells and the surrounding environment utilising various materials, including both synthetic and natural polymers^9^,^19^. However, using this approach and the application of positively charged polymers in direct contact with cells can be detrimental to cell survival and functionality^13^. Hence several groups have demonstrated the effect of modifying polycations with different materials, including poly(ethylene) glycol and phosphoro-choline to reduce their toxicity and inflammatory responses^19^,^20^,^21^. Herein, we chose PLL and ALG as the cationic and anionic polymers, respectively. However, as a novel approach to reduce the toxicity of PLL, cells were first suspended in 100 mM of CaCl_2_, which enhanced the formation of a layer of ALG prior to the addition of PLL on the cell’s surface. Results from live/dead staining suggested that the coating approach used in this study, enhanced cell survival by 25% in comparison with the conventional LBL procedure after being coated for 8 days (Fig. 3c). Also, a possibility for the clusters of dead cells observed in Fig. 3b might be secretion of harmful chemicals, associated with dead cells.

Furthermore, pre-coated cells were able to maintain higher metabolic activity compared with MIN-6 spheroids coated with an initial layer of PLL (Fig. 3D). Importantly, these studies confirmed that suspending cells in 100 mM of CaCl_2_ during the pre-coating step did not induce harmful effect on cell viability, hence it was concluded that with the described coating approach the toxic effect of PLL was reduced without any further need for surface or chemical modification.

The presence of polyelectrolyte layers on MIN-6 spheroids was confirmed by the means of FITC labelled PLL. When FITC-PLL was applied to the last bilayers, clear fluorescent emission was observed from cross sectional images and Z-stack 3D reconstruction of coated spheroid (Fig. 4a,b,c), suggesting the existence and successful deposition of polymeric layers on the surface of cell spheroids.

Although maintaining a high metabolic activity and survival rate of the encapsulated cells could increase the longevity of transplanted cells, complete success in pancreatic islet implantation relies upon efficient immunoisolation of the cells from recipient immune responses. In this regard, to gain a better insight into the coating approach established in this study, both coated and non-coated cells were exposed to FITC-mouse antibody. The images from spheroids were captured through the Z-axis using confocal microscopy (Fig. 5). Observation showed a notable prohibition of FITC antibody from attaching to MHC class II antigens on the surface of coated beta-cells in comparison to the non-coated cells.

Previous studies have shown that release of the three main pro-inflammatory cytokines (IL-1β, TNF-α, and IFN-γ) from activated T-cells and macrophages could be responsible for dysfunction of beta-cells by inducing over expression of inducible nitric oxide synthase (iNOS)^22^,^23^. In the present study, we evaluated whether the coated cells could prevent the diffusion of cytokines to MIN-6 spheroids. Figure 6a, emphasised that deposition of pre-coating layer and four bilayers of PLL/ALG was effective in inhibiting cytokine damage and hence increasing cell viability compared with naked spheroids.

We further demonstrated the MIN-6 spheroid’s ability to sense the changes in glucose concentration (2 Vs. 20 mM) during the GSIS assay. For both coated and non-coated cells, increasing glucose concentration from basal to stimulatory, significantly increased the amount of insulin secreted by cells (Fig. 6b). Hence the data showed that suspending cells in CaCl_2_ solution during the pre-coating step followed by the addition of 4 bilayers of PLL/ALG, did not affect MIN-6 cells in terms of insulin release.

In conclusion, we developed uniform sized and shaped MIN-6 spheroids using an agarose construct with the advantage of forming robust 3D cell spheroids that may be easily harvested. The feasibility of the reported novel calcium pre-coating approach to enhance cell survival and functionality of pancreatic beta-cells was demonstrated using various assays. Use of this pre-coating step was found to be an effective way for preserving MIN-6 cellular metabolism and viability compared with conventional PEM coating procedures. Similar to non-coated cells, coated pancreatic beta-cells allowed a rapid response to external glucose changes. Promisingly, incorporation of this pre-coating step followed by four bilayers of ALG and PLL obstructed large immunological molecules as well as cytokines compared to naked spheroids. In summary, this novel coating method was shown to be a promising immunoisolation therapy which may be utilised for the treatment of type 1 diabetes and potentially other endocrine diseases.

## Methods

### Cell culture

MIN-6 cells (AddexBio, San Diego, USA) were cultured in Dulbecco’s modified eagle medium (DMEM, 4500 mg/L glucose, Sigma, UK) supplemented with 15% fetal bovine serum (FBS, Sigma, UK), 2 mM L-glutamine (Sigma, UK) and 0.05 mM 2-mercaptoethanol (Sigma, UK)^22^. Since mouse pancreatic beta-cells have limited ability to tolerate oxidative stresses, 2-mercaptoethanol was added to the cell culture medium to aid in maintaining a reducing environment; limiting toxic oxygen radicals. All cultures were incubated at 37 °C with 5% CO_2_ until they reached 70–80% confluency. Culture medium was changed every other day to enhance cell growth and viability.

### Seeding beta-cells within micro-wells

Micro-molds (Sigma-Aldrich, UK) were used for casting 3-dimensional (3D) culture plates by adding 500 μL of 2 w/v% agarose solution into each mold. The final transparent agarose culture plate with 400 μm diameter micro-wells (number of micro-wells per mold: 256) were removed from the mold after being cooled. Cells were first trypsinized with trypsin-EDTA (Sigma, UK), and counted with a haemocytometer. MIN-6 cells suspended in supplemented DMEM, at a density of 5 × 10^5^cells/200 μL, were seeded on top of the concave agarose micro-wells. Spheroid formation was observed daily using an inverted microscope (Olympus Co., Germany), and formed spheroids were used for further studies after being cultured for 4–5 days (Fig. 1a).

### Size analysis of MIN-6 spheroids

The size of the MIN-6 cell spheroids formed in micro-wells were compared with the same concentration of cells (2.5 × 10^6^ cells/mL) seeded on flat-bottomed non-attachable culture plates. The average diameters of the two groups were determined on day 7 of culture from thirty randomly selected samples, which were observed using a light microscope (Olympus Co., Germany). Quantitative data was determined using Image J software (NIH, Bethesda, MD, USA).

### Coating MIN-6 spheroids with multi-polyelectrolyte layers

After 4–5 days of culture, cell spheroids were retrieved from the agarose micro-wells using a plate shaker (Grant-bio, PMS-1000i, UK) (450 rpm). Solutions of alginate (1 mg/mL) (ALG, medium viscosity (≫20,000 cP, Sigma, UK) and Poly-l-lysine (1 mg/mL) (PLL, MW: 30,000–70,000, Sigma, UK) were prepared in deionised water and Phosphate buffered saline (PBS, Fisher Scientific, UK), respectively. All solutions were sterile filtered using a 0.22 μm syringe filter (Millipore, USA) and UV sterilised overnight. Contrary to the conventional layer-by-layer deposition, before starting the coating process cell spheroids were first suspended in 200 μl of CaCl_2_ (100 mM). After washing with PBS, 200 μL of alginate was added to minimise the potential toxicity of direct contact between cells surface and cationic polymer (PLL). Alternating layers of PLL and alginate were then applied according to conventional LBL methods^24^,^25^. Briefly, 200 μl of PLL was added to the pre-coated spheroids, after 4 min deposition time, cells were washed twice in 500 μl PBS to remove any unbound PLL, followed by the addition of the anionic polymer (alginate) with the same procedure. The last two steps were repeated to achieve the desired number of layers. Finally, cells were washed three times with DMEM and were incubated at 37 °C for further studies.

### Fluorescent labelling of PLL

To prepare the fluorescently labelled PLL, 0.1 mg of Alexa Fluor 647NHS ester (Succinimidyl Ester) (Thermo Fisher, UK) was dissolved in 1 mL of dimethyl sulfoxide (DMSO, Sigma, UK). 100 μL of the dye was added to 1 mL of PLL stock solution with a concentration of 2 mg/mL. The solution was incubated at room temperature for 6 h under continuous stirring. To remove the excess dye, the solution was then dialyzed for 24 h against deionised water. The final solution was then kept in the dark at 4 °C until needed.

### Assessment of cell viability and metabolic activity

Viability of coated MIN-6 cells using different coating deposition sequence was evaluated using a live/dead staining kit. Briefly on the day of experimentation, 1 μL of calcein acetoxymethylester (Calcein AM (662 Da), indicator for live cells, Invitrogen, USA) and 5 μL of propidium iodide (PI (668 Da), indicator for dead cells, Invitrogen, UK) were added to a 200 μL suspension of coated spheroids in cell culture medium. Cells were incubated in the dark at 37 °C for 30 min. Stained cells were then visualised using confocal laser scanning microscopy (Olympus FV1000, Multiple Ar laser, Germany), and images were analysed using ImageJ software (NIH, USA).

The metabolic activities of coated and non-coated (control) MIN-6 cells were assessed using the Alamar Blue cell proliferation assay (Thermo Fisher, UK). The assay reagent reduces from a non-fluorescent blue colour to a fluorescent red form, during cellular metabolism. Hence, the amount of assay reagent reduction is proportional to cell number (presuming equal metabolic activity). Briefly, 20 μL of Alamar Blue reagent were added to 200 μL of cell spheroids suspension in DMEM, followed by incubating for 4 h at 37 °C. The fluorescence was then read (590 nm emission and 560 nm excitation) using a microplate reader (GloMax-Multi+, Promega, USA). The results were reported as percentage reduction between the coated and the control sample.

### Immunofluorescent staining

After 5 days in culture, MIN-6 spheroids were fixed with 10% formalin (Sigma, UK). Samples were then blocked in PBS solution containing 2% bovine serum albumin (BSA, Sigma, UK) and 0.2% Triton X-100 (Sigma, UK) for 1 h. After washing in PBS, fixed cells were stained with Actin-red 555 (F-actin Probe, Life Technologies, UK) for 30 min, followed by several washing steps with PBS. Cell nuclei were also counter-stained using DAPI (1 μg/mL) (Sigma, UK). 3D visualisation of the stained spheroids was undertaken using confocal microscopy (Laser 405 nm: DAPI, Laser 543: Actin-red 555).

### Antibody exclusion assay

The permeability of the coated spheroids against large immunologically relevant molecules was evaluated using FITC labelled anti-mouse MHC class II (eBioscience, UK). Briefly, cell spheroids with and without coating layers were prepared as described previously. Fifteen MIN-6 spheroids were randomly selected from each group, and 100 μL of 2% BSA and Hanks’ balanced salt solution (HBSS, blocking buffer, Sigma, UK) were added to the samples. Cells were incubated for 15 min at 37 °C with 1 μL of FITC- antibody. Samples were then washed twice with HBSS and 0.1% Tween-20 to remove any excess antibody. The coated and non-coated spheroids were observed using confocal laser scanning microscopy with an argon laser.

### Treating MIN-6 cells with cytokines

Groups of coated and non-coated spheroids were treated with a mixture of three cytokines at a final concentration of Interleukin-1β (IL-1β, Sigma, UK) (5 ng/mL), mouse tumour necrosis factor-α (TNF-α, Gibco, Life Technology, UK) (10 ng/mL), and mouse interferon-ϒ (IFN-ϒ, Gibco, Life Technology, UK) (100 ng/mL) in DMEM. All treated samples were incubated overnight at 37 °C. The viability of the treated spheroids was measured using the Alamar Blue assay as described previously, and data were normalised to 1000 cells.

### Glucose-stimulated insulin secretion (GSIS) assay

To evaluate the function of MIN-6 cells, the amount of insulin secreted from non-coated and coated cells in response to changes in glucose concentration was measured. Briefly, samples were washed and pre-incubated in Krebs-Ringer bicarbonate buffer (KRBH: 125 mM NaCl, 1.2 mM MgSO_4_, 1.2 mM CaCl_2_, 22 mM NaHCO_3_, 10 mM HEPES, 1.19 mM KH_2_PO_4_) + 0.1% BSA with glucose concentration of 2 mM for 2 h. Then, samples underwent a static incubation for 1 h with low (2 mM) glucose concentration in KRBH + 0.1% BSA followed incubation for a further hour in high (20 mM) glucose concentration KRBH + 0.1% BSA as the stimulator. For each incubation period the cultured supernatant was collected for insulin assay and diluted to the appropriate range based on the assay standard curve. The secreted insulin was measured using an Enzyme-linked immunosorbent assay (ELISA) kit (Millipore, UK), and the colorimetric reaction was quantified using a plate spectrophotometer (GloMax-Multi + Mictoplate Multimode reader, Promega, USA) at a wavelength of 450 nm.

### Statistical analysis

The student’s t-test, assuming equal variance, was used to identify any significant differences between pairs of groups. One-way analysis of variance (ANOVA) was used to identify any significant difference between the means of independent groups. Furthermore, a Bonferroni post-hoc test was performed with ANOVA to find means that were significantly different within groups. A P-value < 0.05 was determined as significant. Results are presented as mean ± standard deviation unless otherwise stated.

## Additional Information

**How to cite this article**: Nikravesh, N. *et al*. Calcium pre-conditioning substitution enhances viability and glucose sensitivity of pancreatic beta-cells encapsulated using polyelectrolyte multilayer coating method. *Sci. Rep.* **7**, 43171; doi: 10.1038/srep43171 (2017).

**Publisher's note:** Springer Nature remains neutral with regard to jurisdictional claims in published maps and institutional affiliations.
